# [Corrigendum] Alteration of the Wnt/GSK3β/β-catenin signalling pathway by rapamycin ameliorates pathology in an Alzheimer's disease model

**DOI:** 10.3892/ijmm.2026.6003

**Published:** 2026-09-28

**Authors:** Jingfei Chen, Zhimin Long, Yanzhen Li, Min Luo, Shifang Luo, Guiqiong He

Int J Mol Med 44: 313-323, 2019; DOI: 10.3892/ijmm.2019.4198

Following the publication of the above article, a concerned author drew to the authors' attention that they had apparently used an inappropriate antibody in their study: In the Materials and methods section, the authors had reported the use of Abcam's recombinant antibody against plant homeodomain (PHD) finger protein 1 (a.k.a. PHF1; cat. no. ab184951) to probe for the unrelated protein, tau paired helical filaments (PHFs). Based on their search of previous literature, the authors acknowledged that they had made a methodological error in selecting the incorrect antibody; therefore, the western blot assay results shown in Fig. 4A on p. 317 for protein PHF1 should be considered to be invalid.

Given that this issue has come to light, the authors have now purchased the correct and specific antibody targeting phosphorylated tau (p-tau-S404) from ABclonal Viotech Co., Ltd (cat. no. AP1378) and completely repeated the relevant experiments, in strict accordance with the original experimental protocols and conditions. In addition, the authors provided the Editorial Office with the raw data from the repeated experiments, and also proof of purchase of the correct antibody. The corrected version of Fig. 4, now showing the revised data for the PHF1 blots in Fig. 4A together with re-quantified bar charts for these data shown in Fig. 4B, is featured on the next page. Additional changes to the text should also be noted: The text in the Materials and methods section, '*Western blot analysi*s' subsection, on p. 315 (left-hand column, lines 14-15), should read as follows (changes highlighted in **bold**): '…rabbit anti insulin degrading enzyme (IDE) antibody (ab133561), **rabbit anti-PHF-1 antibody (ab184951)**, mouse anti-GSK3β antibody (ab93926)…'. In addition, revisions have also been made to the legend for Fig. 4. The authors confirm that the error made in performing these experiments has not had a significant impact on either the results or the conclusions reported in this study, and all the authors agree with the publication of this Corrigendum. The authors are grateful to the Editor of *International Journal of Molecular Medicine* for allowing them the opportunity to publish this Corrigendum; furthermore, they apologize to the readership of the Journal for any inconvenience caused.

## Figures and Tables

**Figure 4:**
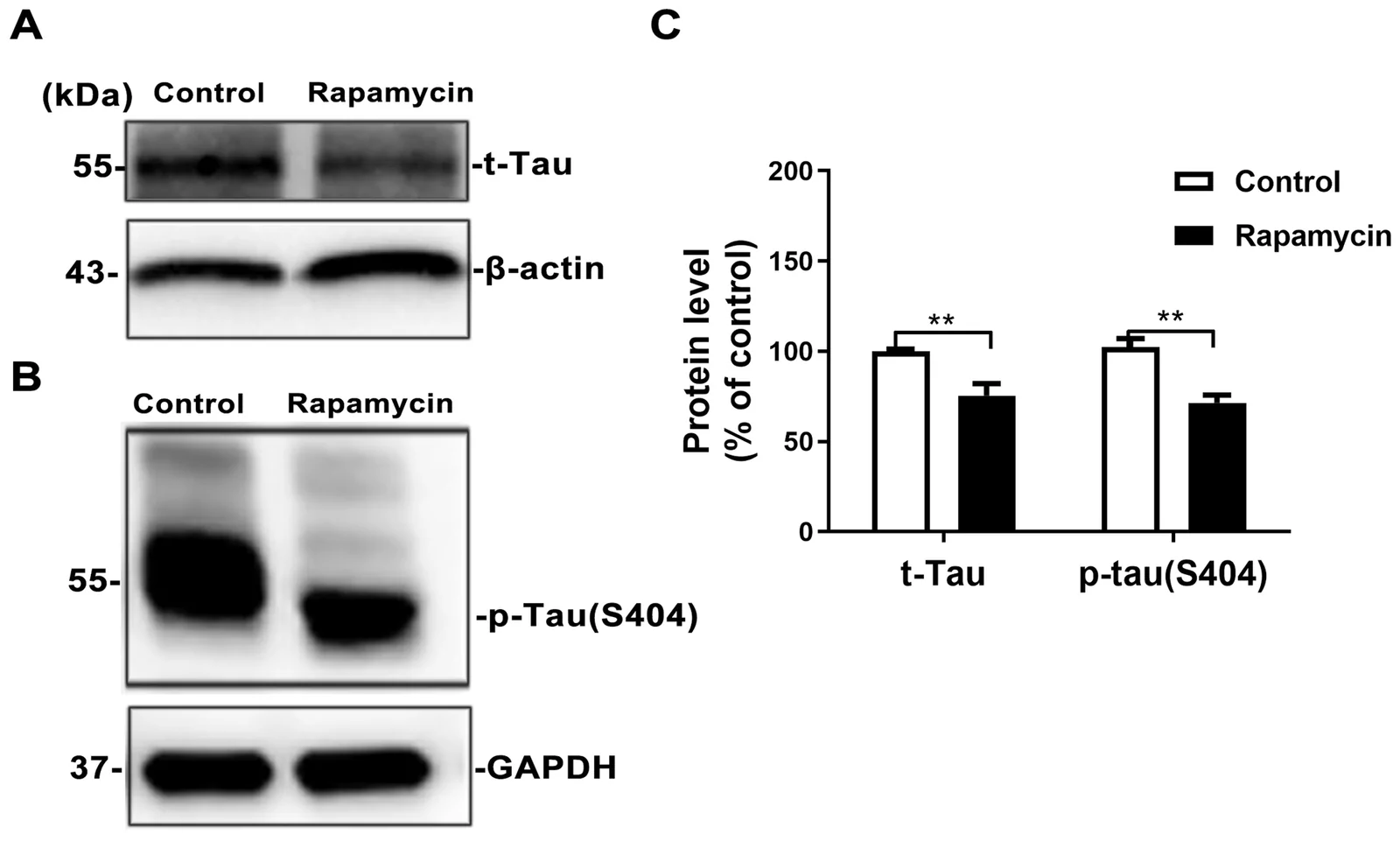
Rapamycin reduced Tau protein levels and hyperphosphorylation. (A,B) Representative western blots of the half brain lysates of 3 mice from the control mouse group and rapamycin-treated mouse group with the indicated antibodies. (C) Quantitative analysis of the blots is shown, and data are presented as the means ± SEM and analyzed by t-test. (^**^P<0.01 compared with the control group). t-Tau, total Tau; p-Tau, phosphorylated Tau.

